# Education Research: Choosing Wisely: A Mixed-Methods Study of Career Choices of Students Completing Advanced Neurology Electives

**DOI:** 10.1212/NE9.0000000000200303

**Published:** 2026-03-30

**Authors:** Brian E. Emmert, Amy A. Pruitt

**Affiliations:** 1 Department of Neurology, Division of Epilepsy, Vagelos College of Physicians and Surgeons, Columbia University, New York; and; 2 Department of Neurology, Perelman School of Medicine, University of Pennsylvania, Philadelphia.

## Abstract

**Background and Objectives:**

Specialty choice is a critical decision for medical students who are often torn between multiple specialties; their final choice affects the trajectory of their careers and their long-term well-being. Understanding the process of this decision is invaluable for mentors, particularly those advising students who indicate a substantial interest in a specialty by electing an upper-level rotation.

**Methods:**

We surveyed 120 students who completed an elective neurology subinternship (Sub-I) between 2013 and 2024 about how they decided among specialties. We asked these graduates to provide narrative descriptions of their decision-making process and advice for current students deciding between specialties. We analyzed quantitative data using chi-square tests and qualitative data by content analysis.

**Results:**

Fifty-nine of 120 graduates responded (49% response rate). Thirty (51%) are currently practicing neurology and 29 (49%) are practicing another specialty. Sixty-four percent changed their premedical school specialty preference during school (*p* < 0.001). More than half (54%) made their final decision during the subinternship (*p* < 0.001), and 63% required a second rotation in multiple specialties. Future neurologists reported greater comfort with uncertainty (*p* < 0.001) and placed higher importance on mentorship than those who chose other specialties (*p* = 0.02). Twenty-eight respondents (47%) had an “epiphany moment” that led to their decision, whereas 31 (53%) did not (*p* > 0.05). The major themes identified in the decision-making process were clinical experience, intellectual curiosity, and the culture/lifestyle of the specialty.

**Discussion:**

This study demonstrates that high-quality subinternships and strong mentorship play essential roles in guiding undecided medical students. Clinical experience, culture and lifestyle of the specialty, and intellectual curiosity are key factors which shape this decision. These insights can help mentors, especially those advising neurology subinterns, provide targeted guidance, supporting students in choosing a specialty that is both personally fulfilling and intellectually rewarding.

## Introduction

One of the most critical choices that medical students make in medical school is their ultimate specialty. The gravity of this choice is incalculable, shaping the trajectory of their entire lives. This is a complex decision, involving a nuanced calculus of multiple factors, each weighted differently by individual students.^1,2^ For some, specialty selection follows a deliberate, structured thought process, while for others, it occurs through an “epiphany moment,” where a specialty suddenly feels like a calling.^3,4^

Medical educators play a crucial role in this decision as advisors, teachers, and mentors, guiding students toward a fulfilling career, while they discern their specialty.^5^ Educators, who differ in mentoring experience, use diverse methods including personal experiences, formal training, and psychological techniques. These often prove effective—many students choose a specialty early, remain in it throughout their careers, and experience personal fulfillment.^6,7^ Yet some students remain undecided even after clerkships, requiring additional electives and subinternships (Sub-Is) to discern a specialty. Of greatest concern are the students who later feel dissatisfied in their chosen field and who might have benefited from more explicit, evidence-based guidance during their medical school years.

To better support students, it would be useful for educators to understand why students choose certain specialties, what factors promote fulfilling careers, and why some choices prove less satisfying. Robust research has explored how the preclinical course and clerkships influence specialty selection, as well as factors associated with pursuing a career in neurology.^8,9^ For example, previous studies show that early clinical exposure, strong pre-clerkship experiences, and meaningful interactions with neurologists are linked to choosing a career in neurology.^8^ Similarly, students who rated their preclinical neuroscience and clerkship experiences highly were more likely to enter the field, whereas those who perceived a poor “personality fit” were less likely to do so.^9^

This understanding is particularly important when advising students who demonstrate strong interest in a specialty by selecting an upper-level Sub-I elective, a topic that remains understudied in medical education literature. Enhancing mentorship for these students may help foster prosperous, fulfilling, and sustainable careers across all medical specialties. To address this gap, we focused our efforts on students who enrolled in an upper level Sub-I to better understand the decision-making process of those who finalize their specialty choice later in medical school.

To frame our investigation, we propose a conceptual model in which specialty choice emerges from the interplay between an internal decision-making process (i.e., rational deliberation vs intuitive epiphany moment) and external influences, such as mentorship and experiential learning, grounded in the dual process theory of decision-making.^10^ Various conceptual frameworks have been applied to medical students' specialty decisions. For instance, social dominance theory suggests that students are drawn to specialties based on prestige and hierarchy, while the possible selves the framework posits that students imagine themselves in hypothetical future careers.^11,12^ However, these frameworks are limited—social dominance is too narrow in scope, and the “possible self” framework assumes most students follow a bottom-up, constructivist approach to decision making.

By contrast, the dual process theory of decision making posits 2 distinct systems: a bottom-up deliberate process (system 1) and top-down, intuitive, and rapid process (system 2). We refer to the latter process as the epiphany moment to emphasize the contrast between rational deliberation and intuitive insight—an experience frequently described by students whom we mentor. This framework shaped our analysis of how students in the neurology Sub-I made their specialty decisions.

This study surveys all former students, now practicing physicians across multiple specialties, who completed the advanced neurology Sub-I elective at the University of Pennsylvania over a 12-year period. The Sub-I elective experiences included rotations on the consult, ward, and neurointensive care services. Sub-Is met with the course director at the beginning and end of rotation and spent the rest of their rotations with the residents and attendings, without a formal mentorship program. We asked participants to reflect upon how they chose their specialty, including the factors they weighed, the timing of their decision, and whether it emerged from a deliberate thought process or an epiphany moment. We also invited them to share advice with future students facing a similar decision of choosing between different specialties. This study aims to characterize the timing, methods, and factors underlying specialty selection among former neurology Sub-I students to arm mentors and advisors who advise neurology Sub-I students with evidence-based insights for guiding undecided medical students.

## Methods

We conducted a mixed-methods cohort study using a convergent parallel design. We selected this approach to provide a more comprehensive understanding of our research questions. The qualitative component allowed for an in-depth exploration of participants' thought processes, while the quantitative component enabled a broad evaluation of other factors, which may have influenced their decision. Survey questions were developed based on faculty experience mentoring medical students and previous studies of career decision-making.^13-15^ Researchers used a pragmatist paradigm in designing and planning the survey and analysis.^16^ This paradigm was selected to allow for flexibility in data collection and analysis and to emphasize the practical applications of the findings. Within this framework, the qualitative component was informed by the interpretivist perspective, focusing on participants' subjective reflections and interpretations regarding specialty choice. Quantification of qualitative themes was used to support integration with the quantitative findings, consistent with our pragmatist approach. Three senior neurology faculty with extensive experience in medical education reviewed the survey for accuracy and clarity. A pilot run was conducted with 3 former students not included in the final cohort to ensure completeness and comprehensiveness. The survey was administered through Qualtrics (Seattle).

### Reflexivity and Trustworthiness

Throughout the analysis, the research team engaged in reflexivity to enhance trustworthiness. The research team included physicians and career medical educators with personal experience in navigating career decision-making and choosing between specialties, which had the potential to shape interpretations. To address this, the team documented reflexive memos during coding about reactions, assumptions, and potential biases, which were then discussed at team meetings.

B.E. Emmert is a career medical educator with formal fellowship training in medical education, which included research methods, and is an epileptologist. B.E. Emmert did not teach or mentor any of the participants. A.P. Pruitt is a career medical educator, neurologist, and longstanding clerkship and subinternship director. A.P. Pruitt served as the clerkship and subinternship director for all participants.

Credibility was supported through independent coding by all team members, followed by discussion and consensus building to refine codes. Dependability was enhanced by maintaining a systematic analytic approach and documentation of analytic decisions. Triangulation across qualitative and quantitative components of the mixed-methods design strengthened confidence in the findings.

### Standard Protocol Approvals, Registrations, and Patient Consents

This project was deemed intitutional review board (IRB) exempt because it posed no more than minimal risk and was sufficiently anonymized (IRB number 855792).

We used a targeted sampling strategy by which we identified 122 former internal Perelman School of Medicine students who took a neurology Sub-I (Neurology 3000) course between 2013 and 2024 using course enrollment and school records. External Sub-I rotators were excluded given the absence of consistent records of former external rotators. We supplemented incomplete contact information with our personal records or Doximity when needed. Of these, 120 students were contacted; 2 lacked any contact information. Survey invitations, which included a brief description of and reason behind the research, were distributed through e-mail or Doximity Messenger. The description included a description of who the investigators were and what their roles were, including A.P. Pruitt as their clerkship and subinternship director. We set a predetermined minimum response rate goal of 40% based on response rates of previous survey studies of former students/residents.^17,18^ The initial solicitation received 39 responses; a reminder e-mail 1 week later yielded 20 additional responses.

Quantitative responses were initially compared between participants who pursued neurology and those who did not. Chi square tests were used for categorical variables, and Wilcoxon rank-sum tests were used for continuous variables (data were not normally distributed). Post hoc analyses compared neurology respondents with respondents in psychiatry, medicine, and radiology using a chi square analysis. Analyses were performed using Stata SE 13.1 for Windows (Stata Corp, College Station, TX).

Open-ended survey responses asking participants to (1) describe their epiphany moment in specialty choice (if applicable) and (2) provide advice for students deciding between neurology and another specialty were analyzed using qualitative content analysis. Responses were transcribed, divided line-by-line, and deidentified, using Microsoft Excel. The research team reviewed all responses to familiarize themselves with the content. We then generated codes inductively to allow themes to emerge naturally from the data. Standardized codes were agreed upon collaboratively and applied to each line. Coder reliability was ensured through discussion and resolution of any discrepancies. Related codes were grouped into broader themes representing the major concepts influencing the decision-making process. The frequency of each theme was calculated to quantify their prevalence across the cohort, allowing integration of both narrative depth and quantitative description, using the Pearson χ^2^ test for independence, at time of analysis.

### Data Availability

Data not provided in the article because of space limitations may be shared (anonymized) at the request of any qualified investigator for purposes of replicating procedures and results.

## Results

### Response Rate and Demographics

Of the 120 eligible former students who completed a Sub-I in neurology, 59 responded (49%). Half (51%, n = 30%) are practicing neurologists; the remainder entered other specialties, most commonly medicine (n = 8%, 14%), radiology (n = 5%, 8%), and psychiatry (n = 5%, 8%). Other specialties included dermatology (n = 1%, 2%), emergency medicine (n = 1 2%), and neurosurgery (n = 3%, 6%) (Table 1). Thirty-three respondents (56%) reported having a family member in medicine, with no difference between neurologists and nonneurologists (*p* = 0.68). To preserve anonymity, additional demographic information (i.e., age and sex) was not solicited, given the small number of students who take the neurology Sub-I yearly.

**Table 1:** Distribution of Survey Respondents by Specialty

	N (%)
Neurology	30 (51)
Nonneurology specialties	29 (49)
Radiology	5 (8)
Psychiatry	5 (8)
Medicine	8 (14)
Ophthalmology	2 (3)
Radiation oncology	1 (2)
Neurosurgery/pediatric Neurosurgery	3 (5)
Pathology	1 (2)
Emergency medicine	1 (2)
Dermatology	1 (2)
Out of Practice/pharmaceutical	2 (3)

The table demonstrates respondents in neurology vs nonneurologic specialties. There is no difference in the frequency of responses from neurologists and nonneurologists.

### Specialty Choice and Timing

Before medical school, 56% (n = 33) expressed interest in neurology. Those who expressed interest in neurology before medical school (n = 33) were not more likely to pursue a career in neurology (n = 20, *p* = 0.09). Among those without premedical interest in neurology, the majority (n = 16%, 62%) expressed interest in internal medicine. Overall, 64% of total respondents (n = 38) changed their anticipated specialty during medical school, with no significant difference between future neurologists (n = 18) compared with other specialties (n = 20, *p* = 0.47). During medical school, 34% (n = 20) decided between medicine and neurology and 25% (n = 15) debated among several specialties. Only 14% (n = 8) were set on a specialty at the time of the Sub-I.

Most respondents (58%, n = 34%) made their final specialty decision during their Sub-Is, significantly more than those who made the decision in the clerkship (n = 18, 31%), before medical school (n = 5%, 8%), or during the preclinical course (n = 1%, 3%) (*p* < 0.001). Timing of the final decision did not significantly differ between neurologists (n = 16/30) and non-neurologists (n = 18/29, *p* = 0.25) or among the proportion of neurologists and those in medicine (n = 7/8, *p* = 0.34), psychiatry (n = 2/5, *p* = 0.83), or radiology (n = 4/6, *p* = 0.70). Thirty-seven respondents (63%) reported requiring a second rotation to decide. The proportion of neurologists who needed a second rotation (n = 15/30) was significantly smaller compared with other specialties (n = 22/29, *p* = 0.04), whereas the proportion entering internal medicine (n = 8/8) more often required a second rotation compared with those who went into neurology (*p* = 0.04).

### Factors Included in Decision-Making

Most respondents (80%, n = 47%) had prior neuroscience experience before medical school, defined as research experience, an undergraduate major or minor in neuroscience, shadowing in neurology, or have taken a course in neuroscience. No significant differences were observed between neurologists and nonneurologists overall or by individual type of experience (*p* = 0.95).

Participants rated the relative influence premedical school, preclinical, clerkship, mentorship, patient case, research, and family experiences on their decision to pursue their specialty. The distribution of responses is tabulated in Table 2. Overall, there was no 1 factor which was weighted more heavily than any other between those in neurology and other specialties (all comparisons *p* > 0.05), with the exception of mentorship, which was significantly more influential for future neurologists (16/29 noted a great deal of influence) compared with others (9/26 noted a great deal of influence, *p* = 0.02). This pattern was also seen in comparing neurology with psychiatry (2/5, *p* = 0.004) or radiology (0/4, *p* = 0.006), but not for medicine (3/7, *p* = 0.09).

**Table 2:** Factors Which Influenced Specialty Decision

Factor	It deterred me, n (%)	No influence, n (%)	Some influence, n (%)	A lot of influence, n (%)	A great deal of influence, n (%)
Premedical school experiences	1 (2)	15 (25)	18 (31)	10 (17)	15 (25)
Preclinical experiences	4 (7)	8 (14)	33 (56)	9 (15)	4 (7)
Clerkship experiences	2 (3)	1 (2)	6 (10)	23 (39)	27 (46)
Patient cases	0 (0)	2 (3)	11 (19)	19 (32)	27 (46)
Research	0 (0)	11 (19)	18 (31)	12 (20)	16 (27)
Family experience	0 (0)	23 (39)	18 (31)	5 (8)	5 (8)
Mentorship	0 (0)	5 (8)	10 (17)	15 (25)	25 (42)

Participants rated the relative importance of various factors affecting specialty choice. Overall, no single factor was rated significantly higher than others. Percentages for family experience and mentorship do not sum to 100% as not all respondents reported prior family experience or mentorship.

Participants also compared the influence of specific specialty attributes on career choice (i.e., inpatient vs outpatient, acute vs longitudinal, critically ill vs stable patients, and procedural vs nonprocedural work, certainty vs uncertainty about a diagnosis). Only “comfort with uncertainty about a diagnosis” had a significant influence on a group of students; neurologists reported it as a more influential factor (median 3.5 interquartile range (IQR) 3–4, on 5-point scale) on career choice compared with nonneurologists (median 3, IQR 3–4, *p* < 0.001).

### Epiphany Moments

Twenty-eight respondents (47%) reported a distinct epiphany moment in choosing a specialty, evenly split between those who did and did not go into neurology (n = 14%, 24% for each group, *p* = 0.92). Analysis of these 28 respondents' descriptions identified 5 main themes: clinical experience, intellectual curiosity, pathology and neurologic examination, culture and lifestyle of specialty, and mentorship. Clinical experience was the most frequently cited driver, followed by intellectual curiosity (Table 3). Comparison of theme distribution between neurologists and nonneurologists revealed no significant differences (*p* = 0.49), and post hoc comparisons with radiology, medicine, or psychiatry were similarly nonsignificant (*p* > 0.27 for all).

“I had never considered it seriously before then, but for the rest of the year I kept comparing everything back to neurology.”

**Table 3:** Representative Quotations From Respondents Describing Their Epiphany Moment

Theme	Specialty	Quote
Clinical experience	Radiology	“I realized during clerkships I didn't really like patient facing specialties.”
	Neurology	“I had never considered it seriously before then but for the rest of the y I kept comparing everything back to neurology.”
	Neurology	“I will always remember my two main patients that I cared for on the Neurology ward service during my clerkships.”
Culture and lifestyle	Neurology	“I felt like I found my people.”
	Neurology	“I felt that Neurology was more my tribe.”
	Medicine	“I felt I didn't fit into the neuro culture.”
Mentorship	Neurology	“I was on rounds on ward with Dr. X and watched him localize a myelopathy with his little orange hammer and then wax poetic about reflexes and I thought it was super cool.”
	Neurology	“It was really Dr. P. and Dr. P's direct encouragement that led me to neurology.”
Pathology and examination	Neurology	“I loved the problem solving, the emphasis on physical exam over diagnostic testing.”
	Psychiatry	“I realized that did not enjoy bread and butter neurology.”
Intellectual curiosity	Psychiatry	“As I was preparing to submit residency applications, it was clear that psychiatry residency would offer me much more dedicated research time and allow me to pursue a robust research career with a strong tilt towards basic science.”
	Emergency Medicine	“I realized that I could actually do everything in emergency medicine. I'd always be learning all the other specialties.”
	Neurology	“Neurology was the best way to combine my research interests with a clinical career.”

Several themes emerged from participants' descriptions, and example quotations for each theme are provided by respondents across different specialties.

### Advice to Students

All respondents' responses were analyzed when asked to give advice to students when deciding between neurology and other specialties. The same 5 major themes appeared, although culture and lifestyle of specialty was most frequent, followed by clinical experience, intellectual curiosity, and pathology (Table 4). There were no significant differences in theme frequency between neurologists and nonneurologists (*p* = 0.38), or in the post hoc comparisons between radiology, medicine, or psychiatry.

**Table 4:** Representative Quotations From Respondents Advising Students Between Neurology and Other Specialties

Theme	Specialty	Quote
Clinical experience	Neurosurgery	“Sign up for rotations in all specialties and speak to mentors!”
	Neurology	“Make sure you really try them both in depth and understand what an attending does all day.”
	Neurology	“To really get a feel for what a career may look like, it's important to explore further beyond the clerkship.”
Culture and lifestyle	Neurology	“Culture fit with other people in the specialty matters”
	Ophthalmology	“I advise students to think about their practice setting (balance of inpatient/outpatient), surgical/procedural or not.”
	Neurology	“Your choice of specialty will define your future colleagues.”
	Medicine	“Who are my people and where do I feel more at home”
Mentorship	Neurology	“Meet people in academia, in the community, and private practice to get a sense of how people practice differently.”
	Pathology	“Speak with mentors at all stages ahead of you in training/practice.”
	Neurology	“Talk with faculty members, not just residents, about their work/life balance and career directions. Residency is temporary, attendingship is more long term.”
Pathology and examination	Neurology	“Look beyond the most interesting cases and consider what will you be treating most often and do you like that.”
	Neurology	“Think about the bread and butter of each specialty they're considering and if those diseases/patient populations are what they find interesting and rewarding to treat.”
	Radiology	“Think about the inpatient/outpatient differences, sick/not-so-sick, procedural aspects (if desired), and also if there is a specific diagnosis/pattern of disease of interest.”
	Medicine	“Think about what you want to treat and think about on a daily basis, not just the rare or exciting cases.”
Intellectual curiosity	Neurology	“I think you want to find a specialty where the majority of the field will continue to constantly peak your clinical and/or scientific interest.”
	Neurology	“Pick what makes you geek out the most.”
	Medicine	“Think about why you went into medicine. What is your absolute favorite thing about medicine, and what is the thing you absolutely cannot do?”
Gut feeling	Neurology	“Listen to your heart and ask yourself does this feel right.”

Several themes emerged from their advice, and example quotations for each theme are provided from respondents across different specialties.

When asked about advice for choosing a specialty that they would remain in for the long term, the same 5 themes also emerged, along with 3 minor themes: gut feeling, thoughtful reasoning, and amenability to change (Table 5). Culture and lifestyle of specialty was the most frequently mentioned theme, followed by intellectual curiosity. Those entering internal medicine or radiology emphasized culture and lifestyle more frequently than neurologists, whereas neurologists underscored the importance of mentorship (*p* = 0.001). Post hoc comparisons with psychiatry revealed no significant difference (*p* > 0.05).

“Try to find a specialty with the most gratifying interactions with patients and colleagues. See if the people in that specialty are people who you’d work well with as colleagues.”

“Pick what makes you geek out the most.”

**Table 5:** Representative Quotations From Respondents Offering Advice for Remining in a Specialty

Theme	Specialty	Quote
Clinical experience	Neurology	“Get as much exposure as you can to the different career possibilities (not only medical specialties, but also types of careers, like clinician vs researcher vs a mix.”
	Psychiatry	“Having diverse experiences will make you a better doctor. Life and interests change and that is good.”
Culture and lifestyle	Psychiatry	“Try to find a specialty with the most gratifying interactions with patients and colleagues. See if the people in that specialty are people who you'd work well with as colleagues.”
	Radiology	“Find a place where you feel at home and see yourself able to sustain for many years to come. Find the people that resonate with you.”
	Emergency Medicine	“If you're struggling to choose, pick a specialty that offers a variety of paths within it.”
Mentorship	Neurology	“Talk to advisors who can speak to the different areas you're considering or who may have gone through similar experiences so you can learn from them.”
	Neurology	“I spoke with mentors, talked to people at conferences, and utilized social media neurology threads.”
Intellectual curiosity	Neurology	“Make sure you are interested both in the patients and the overall intellectual challenge of any specialty you go into. What are the articles or paper you enjoy reading late at night for fun”
	Emergency Medicine	“You will never love the entirety of a specialty, but pick one that is weighted towards the things you truly value.”
	Neurosurgery	“Be drastically honest with yourself”

Several themes emerged from their advice, and example quotations for each theme are provided from respondents across different specialties.

## Discussion

Choosing a medical specialty is daunting, and both students and their mentors lack explicit evidence-based guidelines to focus their decision-making. This decision is particularly complex for students deciding among multiple specialties. This study underscores the role of the Sub-I and mentorship in guiding students and identifies several factors that influence specialty choice at the Sub-I level, namely, culture and lifestyle of the specialty, clinical experience, and comfort with uncertainty. With these data, we provide neurology advisors and mentors with structured guidance on how to lead students as they navigate this complex and consequential decision.

When deciding between specialties, a “second look” at the specialty can be immensely helpful for students. Sub-Is are often viewed by educators as a vehicle for those already decided on neurology to enhance their residency application or deepen their understanding of neurology.^18^ The present cohort suggests that the Sub-I can also play a major role in influencing a student's eventual specialty decision, rather than just solidifying their already established decision. Thus, students often take an elective Sub-I with the very targeted goal in mind of helping to discern if that specialty is the right fit. This underscores the value of high-quality Sub-Is, allowing students to evaluate intellectual interest, clinical work, and the culture of the specialty first-hand. Allocating resources and skilled educators to Sub-I electives can have a critical impact on students' career formation, particularly those who are weighing multiple options.

Understanding this mindset of Sub-I students is essential for both effective mentorship and Sub-I administration. Such insight allows educators to tailor the Sub-I curriculum, set appropriate goals and objectives, and guide advisors to be more intentional when helping students weigh factors relevant to different specialties. Students typically enter their Sub-I having already narrowed their choices to 1 or 2 specialties. In our cohort of neurology Sub-I students, the most common decision point was between internal medicine and neurology. Notably, only a minority of students have already decided on neurology at the start of the rotation. These findings highlight the need to reconsider traditional assumptions about the purpose of the Sub-I, tailoring its goals and objectives beyond specialty-specific education to support students who are actively deciding between 2 specialties.^19^

Mentorship emerged as a key factor in students' decisions in the present cohort, especially for budding neurologists. Skilled mentorship provides guidance, experience, and evidence-based advice to support students as they navigate complex decisions, having the capability to make influential impacts on students choosing between specialties.^20^ Medical schools and neurology departments alike can support this by developing robust structured mentorship programs during Sub-I electives, training mentors, and recognizing their contribution by compensating them for their time and effort.^21-24^ Investing in mentorship at the Sub-I level profoundly influences students navigating complex specialty decisions that affect their career trajectories. With the recognition of the importance of the Sub-I experience, and as a consequence of the information gained from this evaluation, several modifications have been instituted at the authors' institution: (1) the Chair now meets with each Sub-I student to explore career goals and (2) an advanced neurology elective director has been named, who also meets with each Sub-I student, personalizing outpatient experiences for these students and working through clinicopathologic conference individually or in small groups.

Students weighed multiple factors, such as clinical setting, acuity, procedural work, but no single factor, consistently corelated with specialty choice.^13-15^ These factors seemed to have different weights for different students. However, 2 factors were particularly salient for future neurologists: comfort with uncertainty and mentorship. Neurology is filled with uncertainty—about ultimate diagnoses, about prognoses, and about best treatment options. Neurologists reported that comfort with diagnostic uncertainty strongly influenced their career choice. Advisors should discuss this trait with students, particularly those exploring neurology vs another specialty. If students derive fulfilment from landing on a clear diagnosis, prognosis, and management plan, then neurology may not be the best option for that student.

The present cohort suggests that students arrived at a specialty decision through both an epiphany moment and rational discernment, irrespective of the final specialty decision. Clinical experiences were paramount to students experiencing an epiphany moment. For instance, students reported that seeing patients with bread-and-butter pathology of their specialty gave them the realization that this was the patient population and pathology they were passionate about. Similarly, while rotating through other specialties, they realized that they were most attracted to patients with pathologies of their chosen specialty (i.e., the medical complications of multiple sclerosis). Mentors can encourage students to have diverse experiences to cultivate this epiphany. Similarly, advising students who use thoughtful discernment on the aforementioned factors which influence career decisions gives them fodder to dissect in their decisions. As it is not clear who will have an epiphany and who will rationally decide on a specialty, mentors should advise students as if they will be using both methods.

When advising students deciding between specialties, respondents emphasized the culture and lifestyle of the specialty as the most critical consideration, especially for nonneurologists, who often noted that they did not “fit” with the culture of neurology. As social beings, humans strive to “fit in.”^25,26^ All specialties have a microculture, which students will join if entering the field. Much of this culture may not be apparent in the heavily inpatient-weighted experiences in most neurology clerkships. Finding “your people” and aligning specialty choice with personal values, is critical to longevity, improved quality of life, career fulfillment, and reduced burnout in a specialty.^27-30^ Embedded mentors in the specialty during the Sub-I can advise students on the culture and lifestyle of the specialty, where the student already has at least some interest in the specialty's intellectual endeavors, clinical work, and patient populations.

Using these findings, we have developed a mentorship guide composed of important questions which mentors can consider asking students who are deciding between neurology and another specialty (Figure).

**Figure:**
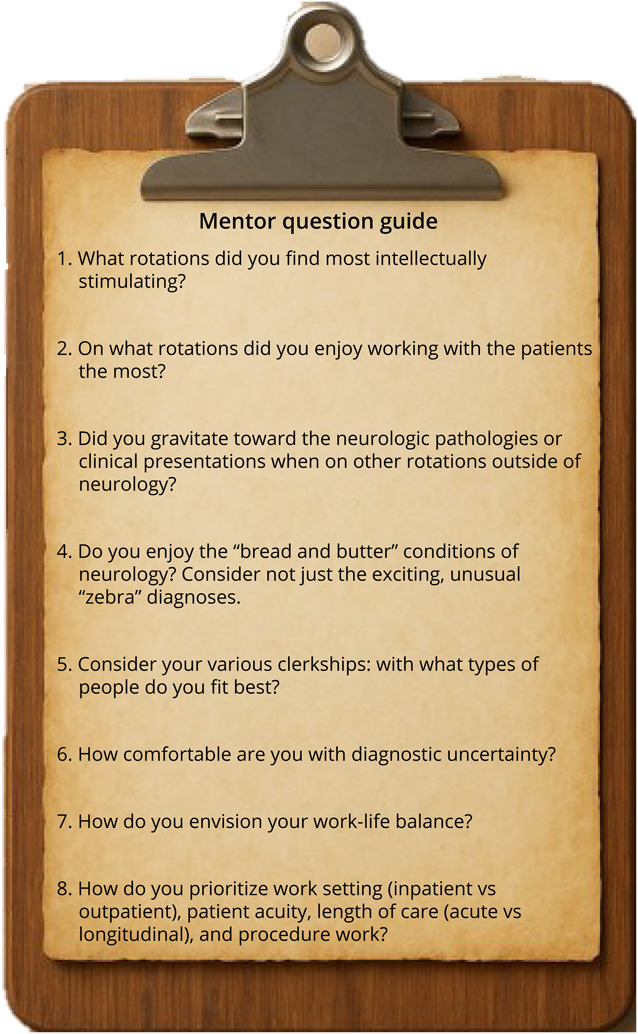
Mentorship Question Guide We have developed a mentorship guide composed of important questions which mentors can consider asking students who are deciding between neurology and another specialty. Combining the answers to these questions can help guide both students and mentors alike toward a specialty.

Mentor guide: Should your Sub-I become a neurologist?

1. What rotations did you find most intellectually stimulating?
2. On what rotations did you enjoy working with the patients the most?
3. Did you gravitate toward the neurologic pathologies or clinical presentations when on other rotations outside of neurology?
4. Do you enjoy the “bread and butter” conditions of neurology? Consider not just the exciting, unusual “zebra” diagnoses.
5. Consider your various clerkship relationships: with what types of people and personalities do you fit best?
6. How comfortable are you with diagnostic uncertainty?
7. How do you envision your work-life balance?
8. How do you prioritize work setting (inpatient vs outpatient), patient acuity, length of care (acute vs longitudinal), and procedure work?

Combining the answers to these questions can help guide both students and mentors alike toward a specialty.

The insights of this cohort can help refine the goals and learning objectives of Sub-Is. Beyond traditional medical knowledge and clinical learning objectives, course directors can include secondary objectives such as “experience the culture of the specialty,” “evaluate the lifestyle associated with the specialty,” or “reflect on the excitement derived from bread-and-butter cases.” Customizing goals in this way can enhance the Sub-I experience for students and better support career discernment.

This study relied on retrospective survey responses, which are subject to recall and availability bias. Any voluntary survey has response bias, and this study also lends itself to social desirability bias. Because this study evaluated a fixed cohort, an a priori power calculation was not performed, and the study may have been underpowered to detect small-to-moderate differences in binary outcomes between groups. Only Penn students who completed an internal neurology Sub-I elective were surveyed, limiting generalizability to all medical students. However, the cohort included many students deciding between multiple specialties, suggesting broader relevance.

Specialty choice is a challenging yet pivotal decision. Students arrive at this choice either through an epiphany moment or rational discernment. High-quality Sub-Is and strong mentorship play essential roles in guiding undecided medical students. Clinical experience, culture and lifestyle of the specialty, and intellectual curiosity are key factors which shape this decision. These insights can help mentors provide targeted guidance, supporting students in choosing a specialty that is both personally fulfilling and intellectually rewarding.
